# Volatile Evolution of Long Non-Coding RNA Repertoire in Retinal Pigment Epithelium: Insights from Comparison of Bovine and Human RNA Expression Profiles

**DOI:** 10.3390/genes10030205

**Published:** 2019-03-08

**Authors:** Olga A. Postnikova, Igor B. Rogozin, William Samuel, German Nudelman, Vladimir N. Babenko, Eugenia Poliakov, T. Michael Redmond

**Affiliations:** 1 Laboratory of Retinal Cell and Molecular Biology, National Eye Institute, National Institutes of Health, Bethesda, MD 20892, USA; olga.postnikova@nih.gov (O.A.P.); Samuelw@nei.nih.gov (W.S.); poliakove@nei.nih.gov (E.P.); 2 National Center for Biotechnology Information, National Library of Medicine, NIH, Bethesda, MD 20894, USA; rogozin@ncbi.nlm.nih.gov; 3 Department of Neurology, Icahn School of Medicine at Mount Sinai, New York, NY 10029, USA; german.nudelman@mssm.edu; 4 Laboratory of Neuropathology Modeling, The Federal Research Center Institute of Cytology and Genetics SB RAS, Novosibirsk 630090, Russia; bob@bionet.nsc.ru

**Keywords:** lncRNA, RPE, evolutionary conservation, bovine lncRNA

## Abstract

Currently, several long non-coding RNAs (lncRNAs) (TUG1, MALAT1, MEG3 and others) have been discovered to regulate normal visual function and may potentially contribute to dysfunction of the retina. We decided to extend these analyses of lncRNA genes to the retinal pigment epithelium (RPE) to determine whether there is conservation of RPE-expressed lncRNA between human and bovine genomes. We reconstructed bovine RPE lncRNAs based on genome-guided assembly. Next, we predicted homologous human transcripts based on whole genome alignment. We found a small set of conserved lncRNAs that could be involved in signature RPE functions that are conserved across mammals. However, the fraction of conserved lncRNAs in the overall pool of lncRNA found in RPE appeared to be very small (less than 5%), perhaps reflecting a fast and flexible adaptation of the mammalian eye to various environmental conditions.

## 1. Introduction

Long non-coding RNAs (lncRNA) are transcripts that do not encode proteins and are longer than 200 nucleotides. According to GENCODEv7, the human genome has 9277 manually annotated lncRNA genes producing 14,880 transcripts [1]. LncRNAs are transcribed by polymerase II and can be polyadenylated and spliced, just as protein coding transcripts. It has been demonstrated that lncRNAs are more tissue-specific and are expressed, on average, approximately 10 times less compared to protein coding mRNAs [2]. While some lncRNAs have described regulatory roles most are functionally uncharacterized [3,4,5,6,7,8,9]. The idea that most lncRNAs are by-products of background transcription, and “simply the noise emitted by a busy machine” [10,11], may be due to the low abundance and poor evolutionary conservation of many lncRNAs compared to protein-coding sequences and small RNAs (e.g., miRNAs and snoRNAs) [12]. However, some lncRNAs have strongly conserved regions [13]. Also, most lncRNAs show reduced rates of substitution and insertion/deletion that suggest purifying selection [6,14,15].

As strong sequence conservation is lacking, identification of lncRNAs at the genome level depends on expression analysis. This makes a complete annotation of the mammalian lincRNome difficult to achieve. By using a variety of approaches, there has been ongoing identification of novel lncRNAs from the transcriptomes of several mammalian taxa [16]. The FANTOM3 project, in particular, has identified >30,000 putative lncRNAs in mouse tissues by full-length cDNA cloning [17,18]. However, an important unsolved issue in these analyses is a lack of a clear boundary between protein-coding and true lncRNA genes. Using the support vector machine technique, the FANTOM3 project transcripts have been separated into coding and non-coding transcripts and thereby applied to estimate how many lncRNAs occur in a mouse. Accordingly, ~14,000 lnc RNAs were newly identified in the FANTOM3 data while the total number of lncRNAs was estimated to be ~28,000 [19]. However, Ji and colleagues showed that many cytoplasmic lncRNA are bound to ribosomes and may be translated to small peptides [20]. Thus, the question of the protein-coding potential of lncRNAs remains open.

Managadze et al. compared the identified human and mouse long intergenic non-coding (linc)RNAs using a new statistical technique to estimate their size and evolutionary conservation [21]. They estimate that human and mouse lincRNAs outnumber protein-coding genes by at least two to one. Furthermore, there is apparent conservation of two-thirds of the lincRNAs between human and mouse, although criteria of orthology were relatively relaxed in this study (100 nucleotide overlap of putative orthologs of human lincRNAs with the mouse lincRNAs and liberal expression cut-off values [21]). This suggests that there exists, as yet, thousands of conserved but uncharacterized functions [21] and is consistent with several other studies that used more stringent criteria of lncRNA conservation [14,22,23,24,25]. Furthermore, analysis of intron-exon structure in lincRNA genes from various mammalian genomes suggests the conservation of introns for over 100 million years [26]. This result suggested that the primary and/or secondary structure of these molecules may be functionally important [26]. Other evolutionary studies suggested that the fraction of conserved lncRNA genes (shared by primates and other mammals) is slightly larger than 20% [23,27], thus various approaches still tend to produce large quantities of evolutionary conserved lncRNA genes in mammalian genomes.

However, conservation of lncRNAs and their gene structure appeared to be tissue-specific. For example, only 18 retinal lncRNAs were found highly conserved between human and mice [28]. Cell type-specific lncRNAs also were shown to be expressed following differentiation of human induced pluripotent stem cells (iPS) into retinal pigment epithelium (RPE) [29]. Currently there are many examples of lncRNAs (TUG1, MALAT1, MEG3 and others) that regulate proper visual function and that may contribute to retinal dysfunction [30]. We decided to extend these analyses of lncRNA genes to the retinal pigment epithelium (RPE). RPE is a monolayer of pigmented cells located between the choroid and retina of the eye. RPE cells are crucial for absorption of the light, transport of nutrients and ions, retinoid visual cycle and phagocytosis of photoreceptor outer segments. These very specific functions of RPE in the eye determine its unique gene expression. We were interested to uncover conserved lncRNAs that could be involved in signature RPE functions and evaluate the fraction of such lncRNAs in the overall pool of lncRNAs found in the RPE. Human eye lncRNAs are not very well characterized, partially because of difficulties with tissue accessibility. Additionally, and compounding this difficulty, human eye layers (choroid, RPE and retina) are notoriously difficult to separate from each other.

Both retina and RPE are derived from neuroectoderm, and they co-differentiate and influence each other. This process must be tightly regulated. Accordingly, lncRNAs are known to be involved in retinal cell fate determination [31], and the development of retinal architecture [32]. In this study, because many eye lncRNAs have tissue-specific expression [33], and it is very difficult to obtain pure human native RPE from donor eyes, we studied predicted lncRNA genes in bovine RPE to understand their functional roles and evolutionary conservation. To predict lncRNAs, we used a tool called FEELnc, which also classifies them with respect to their distance and strand orientation to the closest protein-coding genes. We found a very small set of conserved lncRNAs in the overall pool of lncRNA that could be involved in signature RPE functions conserved across mammals. This may reflect a fast and flexible adaptation of the mammalian eye to various environmental conditions.

## 2. Materials and Methods

### 2.1. Datasets and Cell Cultures

RNA-seq data used in this study are described in Table 1. Cultured human ARPE-19 cells were grown and differentiated as previously described in Samuel et al. [34].

### 2.2. Reconstruction and Analysis of Expression of Novel Bovine Long Non-Coding RNAs (lncRNAs)

First reads from native and cultured bovine RPE were aligned with Star 2.5 (default parameters were used; [36]) to UMD3.1 reference bovine genome. These alignments were submitted to Stringtie (default parameters were used; [37]) to obtain reference-based assembled transcripts for each sample. Consequently, transcripts missing from the reference genome were merged across samples to create a novel putative set of lncRNAs for further classification with FEELnc (default parameters were used; [38]). This step filters all protein coding transcripts, computes the coding potential of candidate transcripts and classifies lncRNAs based on their genomic localization. Also, the statistical model for the coding potential was trained on the bovine NONCODE data (23,696 bovine lncRNAs). All transcripts assembled from single exons were filtered out prior to the analysis. We also filtered out transcripts that were shorter than 200 bp according to the commonly accepted definition of long noncoding RNAs.

### 2.3. Reconstruction of Putative Orthologous Regions in the Human Genome

The genomic coordinates and sequences of exons and introns of reconstructed bovine lncRNA genes were obtained using the BLAT program (https://genome.ucsc.edu/FAQ/FAQblat) applied to the bovine bosTau8 assembly downloaded from the University of California, Santa Cruz, UCSC Table Browser (http://hgdownload.soe.ucsc.edu/downloads.html). Pairwise alignments of these regions in bovine (bosTau8) and human (hg19) genomes were fetched using the Galaxy system [39]. Sequences of putative human orthologous lncRNA exons were extracted from these genomic alignments using an ad hoc program. The genomic coordinates of putative human orthologous lncRNA exons/genes were obtained using the BLAT program (https://genome.ucsc.edu/FAQ/FAQblat) applied to the human hg19 genome assembly downloaded from the UCSC Table Browser (http://hgdownload.soe.ucsc.edu/downloads.html). It should be noted that the prediction of putative human orthologous lncRNA exons/genes depends on the quality of genomic alignments. Unfortunately, there are well known problems with the accuracy of genomic alignments [40] thus we expect that at least some putative human orthologous lncRNA exons/genes will be missed or incomplete.

To control for the possibility of contamination of the lncRNA data set with previously unidentified protein-coding genes, an additional test was performed: the coding potential of lncRNAs was predicted using the SYNCODE program using pairwise alignments of bovine and human lncRNA genes [15,41]. All lncRNAs showing signs of evolutionary conservation similar to protein-coding genes were removed from the lncRNA set following the strategy suggested in previous studies [2,27].

### 2.4. Analysis of Expression of Putative Human lncRNA Genes

A new GTF file for the human genome was created after adding novel putative lncRNAs. We used dbgap RNA-seq data from human RPE/choroid and retina to elucidate how many predicted lncRNA were expressed in native human RPE. Reads were mapped onto the human GRCh38.9 reference genome and counted with “CLC Work Bench”. Differentially expressed transcripts/genes (DEGs) were predicted with DESeq2 (default parameters were used; [42]). Transcripts were considered to be DEGs when fold change >2 and FDR < 0.01.

### 2.5. Confirmation of lncRNA Transcript Sequences

RNA was extracted from bovine RPE and ARPE-19 cultured human RPE cells as previously described. Two sets of cDNA were prepared separately using Quantum oligo dT primer or random hexamers. For all samples no-RT controls were generated and used for polymerase chain reaction (PCR) with all lncRNAs. Since all tested lncRNAs appeared to be polyadenylated, only polyA cDNA was used for PCR. When possible, primers were designed to the 3′- or 5′-most ends of predicted transcripts. Also, short read sequencing was used to design primers to check for fusion presence, lncRNA/untranslated region (UTR), and protein coding genes. PCR products were subjected to gel purification if multiple bands were present. All PCR products were TA cloned in TOPO vector and sequenced. Transcript start and stop sites of human *NEAT1* (*MSTRG.13090.1* and *MSTRG.13090.2*) transcripts expressed in ARPE-19 cells were determined using rapid amplification of cDNA ends (RACE; Clontech) technique according to the manufacturer’s protocol. Novel full length transcript was submitted to Gen Bank with ID MK562403.

## 3. Results

### 3.1. Reconstruction and Analysis of Expression of Novel Bovine lncRNAs

**Figure 1:**
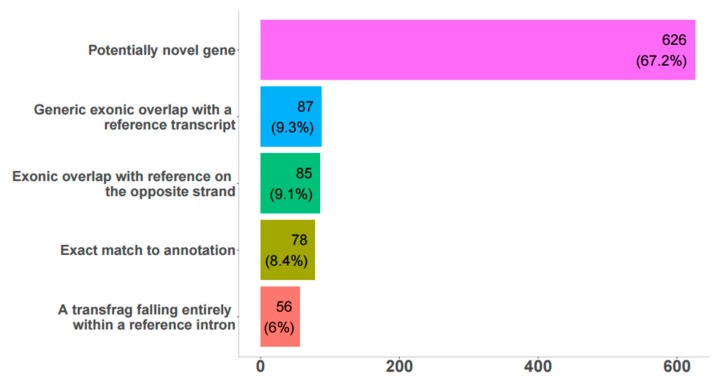
Comparison of detected long non-coding RNAs (lncRNA) to reference genome annotation. Bar graph showing number and percentage of transcripts obtained from FEELnc analysis with low coding potential that belongs to each annotation according to the UMD3.1 reference bovine genome.

The total number of transcripts predicted from FEELnc was 848 (656 genes), all of which were potential bovine RPE lncRNAs (Figure 1, Supplementary File S1.GTF).

Among these, about 67% were potentially novel genes, 8% were an exact match to annotated genes, and another 25% were partially overlapping with transcripts in the reference genomes on the sense or antisense strand. These 848 lncRNAs were classified as genic (157), intergenic (542), and lncRNAs spanning both annotated genes and extending into intergenic space (149), (Supplementary Table S1). 67% (572) of lncRNAs were expressed in native bovine RPE, according to RNA-seq data. In general, there was very good agreement between predicted transcripts in the bovine genome and sashimi plots in IGV (Supplementary Figure S1); 106 and 159 unique differentially expressed (DE) lncRNAs were found between fresh and 4- and 8-week cultured RPE, respectively; 233 DE lncRNAs were common between both time points. Only 23 transcripts had significant hits with the RFAM database (Supplementary Table S2), and some of these have known important biological roles, including MALAT1, TUG1, MEG3, H19, Xist, Six3os1, and NEAT1.

### 3.2. Reconstruction and Analysis of Expression of Putative Orthologous Human lncRNA Genes

In our analysis, 642 gene regions in the human genome with substantial homologies (≥60% identity) were annotated as corresponding to bovine lncRNAs. It was predicted that 818 transcripts potentially could be transcribed from these genes (Supplementary File S2.GTF). There were 698 transcripts longer than 200 bp, with the longest one 8243 bp. We compared this data set with novel lncRNAs described in fetal RPE and induced Pluripotent Stem Cells (iPSCs) [29] and did not find any overlap. This could be explained by the different focuses of the studies. The set of lncRNAs from fetal or iPSC-RPE excludes all known annotated lncRNA. Moreover, adult human native RPE has its own novel set of lncRNA, that are not expressed in fetal or iPSC derived RPE cells.

To determine expression level of lncRNAs in native RPE and retina we used the dataset from Whitmore et al. 2014 [35]. There were 38 regions homologous to bovine lncRNA genes with an average of 20 reads mapped for all RPE samples. These 38 genes we considered to be expressed in native RPE with higher probability. Also, 54 genes were differentially expressed between the human retina and RPE samples. Because we were interested in determining whether lncRNAs are involved in differentiation and maintenance of RPE, we used our bovine data to identify possible candidates. We found 37 potential lncRNAs to be differentially expressed lncRNAs both between RPE and the retina, and between native bovine RPE and cultured bovine RPE cells. To this list we added 12 lncRNAs that were expressed in native RPE and retina at the same level but whose expression level changed when RPE was explanted and cultured. In this way, a list of 49 potentially functional RPE lncRNAs was obtained (Supplementary Table S3). After annotation of these 49 transcripts on the human genome, we found that only 12 of these were true lncRNAs. The remaining 37 overlapped with protein coding genes, usually in the UTR regions. We used PCR analysis to examine 3 lncRNA transcripts that are located within annotated UTR regions of protein-coding genes in the human genome, but not in the bovine genome. Based on the presence or absence of PCR product after amplification with one primer located in the protein-coding gene and the other located in the hypothetical lncRNA from bovine cDNA, only mstrg.14819 could be considered as a lncRNA transcript distinct from the nearby protein-coding transcript. Among 12 conserved differentially expressed lncRNAs, we selected 5 (highlighted in yellow in Table 2), based on their expression levels, to investigate the intron-exon structure of the full transcripts. We chose to focus on these candidates as they were potentially important for development and differentiation of RPE. All were confirmed by PCR to be expressed in native bovine RPE.

### 3.3. Experimental Verification of Novel Bovine and Human lncRNAs.

For experimental confirmation, essential for lncRNA studies, we selected 13 bovine transcripts with expression levels ranging from 0.6 to 400 Transcripts Per Kilobase Million (TPM) in native bovine RPE. All were confirmed as being expressed using quantitative PCR (Q-PCR), with Pearson correlation coefficient R = 0.9 (Supplementary Table S4.). Conserved lncRNAs were in syntenic regions of the genomes, as expected by the parameters of the applied method. One of such regions contains newly annotated NEAT1 and MALAT1 genes in bovine genome (Supplementary Figure S2).

#### 3.3.1. LncRNA Gene: *NEAT1*

**Figure 2:**
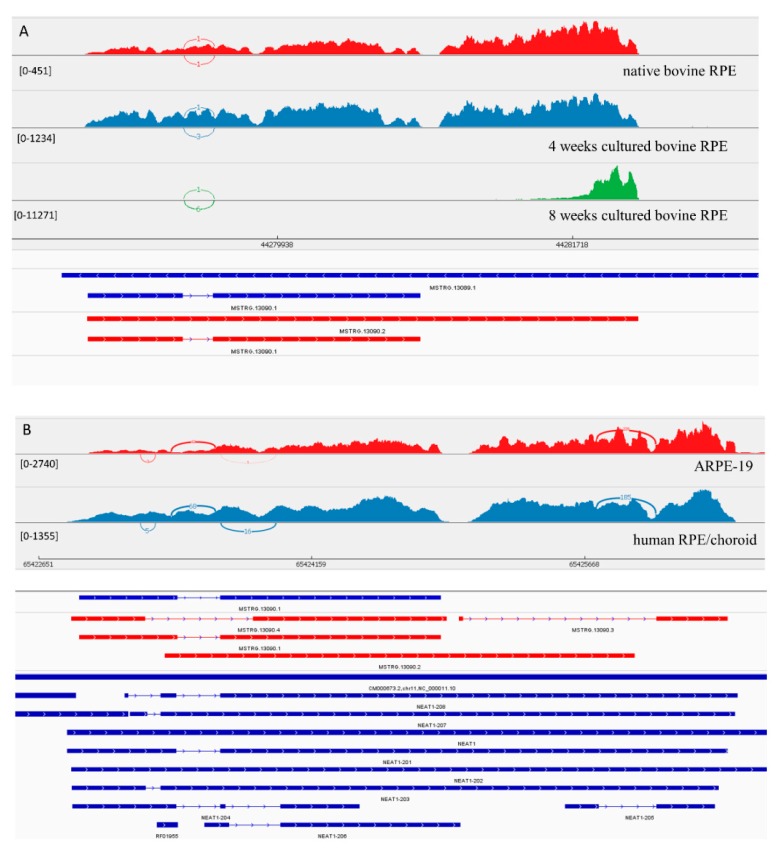
View of *NEAT1* from the Integrated Genome Viewer (IGV). IGV–Sashimi plots showing the read coverage and transcript isoforms. The histograms represent the sum of the aligned sequencing reads along the genome. Numbers in square brackets indicate coverage range. Junction reads are plotted as arcs, and the number of reads aligned to the junction spanning the exons connected by the arc is indicated. (**A**) Sashimi plots from bovine genome; red histogram represents the coverage for native bovine RPE, blue is 4-week cultured bovine RPE, and green is 8 weeks cultured bovine RPE. Below the histograms are shown the predicted isoforms (blue), experimentally verified in this study (red). (**B**) Sashimi plots from human genome; red histogram represents coverage for ARPE-19 cells and blue represents the native human RPE/choroid. Below the histograms are shown the predicted isoforms (blue), experimentally verified in this study (red), and Ensembl genome browser isoforms (blue).

*MSTRG.13090.1*, the homolog of *NEAT1* (Nuclear Paraspeckle Assembly Transcript 1) in bovine RPE, has its expression level increased under culture conditions. *MSTRG.13089.1* is a longer homolog of *NEAT1* (Figure 2). The coverage of this transcript changed in cultured bovine RPE cells after 8 weeks in culture. There was an increased coverage of the 3′ region of the *MSTRG.13089.1* transcript, suggesting the presence of a shorter novel isoform. Also, similar to the human transcripts, we suggest that in native bovine RPE cell these isoforms also were present. Experimentally in ARPE-19 cells, we confirmed the presence of 3 isoforms, that have not been previously annotated in Ensemble.

#### 3.3.2. LncRNA Gene: *MSTRG.5066*

**Figure 3:**
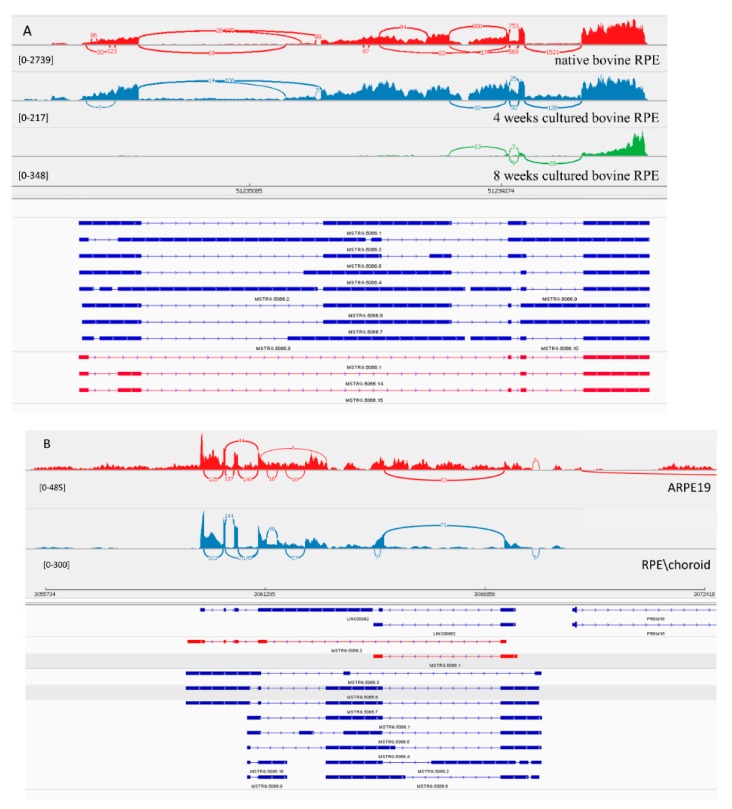
View of *MSTRG.5066* from the Integrated Genome Viewer (IGV). IGV–Sashimi plots showing the read coverage and transcript isoforms. The histograms represent the sum of the aligned sequencing reads along the genome. Numbers in square brackets indicate coverage range. Junction reads are plotted as arcs, and the number of reads aligned to the junction spanning the exons connected by the arc is indicated. (**A**) Sashimi plots from bovine genome; red histogram represents the coverage for native bovine RPE, blue is 4-week cultured bovine RPE, and green is 8 weeks cultured bovine RPE. Below the histograms are shown the predicted isoforms (blue), experimentally verified in this study (red). (**B**) Sashimi plots from human genome; red histogram represents coverage for ARPE-19 cells and blue represents the native human RPE/choroid. Below the histograms are shown the GenBank or predicted isoforms (blue) and experimentally verified isoforms in this study (red).

*MSTRG.5066* is homolog of human *linc00982* gene. Expression of transcripts from this gene were dramatically decreased upon explantation and in primary culture over time. This fact, and the conservation of this lincRNA, suggests a potential functional role of linc00982 in the differentiation process of RPE. For this transcript, 10 isoforms were predicted computationally. In native bovine RPE we could clone only 3 shorter isoforms with 4 and 5 exons (Figure 3A). With such a complicated splicing pattern, Stringtie over-annotated its transcripts. In the human genome, 2 isoforms were confirmed with 5 and 2 exons (Figure 3B). Both matched 100% to previously annotated transcripts in Ensembl. Inexplicably, we could not amplify the transcript from bovine native RPE containing an exon predicted to belong in the middle of the transcript, and there was no PCR product from either the 5′ or 3′ end of the transcript containing this long exon.

#### 3.3.3. LncRNA Gene: *MSTRG.5719*

**Figure 4:**
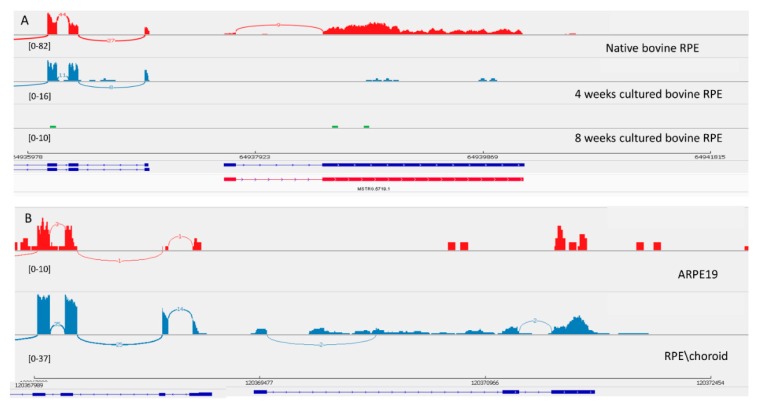
View of *MSTRG.5719* from the Integrated Genome Viewer (IGV). IGV–Sashimi plots showing the read coverage and transcript isoforms. The histograms represent the sum of the aligned sequencing reads along the genome. Numbers in square brackets indicate coverage range. Junction reads are plotted as arcs, and the number of reads aligned to the junction spanning the exons connected by the arc is indicated. (**A**) Sashimi plots from bovine genome; red histogram represents the coverage for native bovine RPE, blue is 4-week cultured bovine RPE, and green is 8 weeks cultured bovine RPE. Below the histograms are shown the predicted isoforms (blue), experimentally verified in this study (red). (**B**) Sashimi plots from human genome; red histogram represents coverage for ARPE-19 cells and blue represents the native human RPE/choroid. Below the histograms are shown the Ensembl genome browser isoform (blue).

Transcript *MSTRG.5719* was originally found in the bovine genome and was experimentally confirmed as a 100% match to the Stringtie prediction. This transcript also decreased in level of expression in culture. Interestingly, *MSTRG.5719* is located next to the splicing factor MSI1 that controls photoreceptor-specific exons in retina [43]. It seems that the exon-intron structure of this transcript is different in the human genome (Figure 4). This transcript is not expressed in ARPE-19 cells, but in the human genome there is an annotated *RPS27P25-202* transcript that is homologous to the bovine transcript.

#### 3.3.4. LncRNA Gene: *MSTRG.1909* (*Linc1833*)

The *MSTRG.1909* transcript located in close proximity to the *SIX3* gene plays an important role in eye development. Expression of this transcript, as well as of *SIX3*, decreased in primary RPE culture compared with fresh native bovine RPE. We were able to confirm only the short isoform in human. This is likely to be an example of a lncRNA gene with a fast rate of evolution of exon-intron structure.

#### 3.3.5. LncRNA Gene: *MSTRG.2517* (*Linc00094*)

*MSTRG.2517* had a similar pattern of diminishing expression in primary culture as *linc1833*, *MSTRG.5719*, and *linc00982*. *Linc00094/BRD3OS-202* is a convergent ncRNA that lies in the promoter region of the *BRD3* gene in the human genome. The *linc00094* isoform *BRD3OS-202* was confirmed as being expressed in ARPE-19 cells by cloning of the full transcript, but the *BRD3OS-205* transcript, coding a small protein of 84 amino acids, was not. We were unable to amplify this transcript from bovine RPE possibly because of its low expression.

## 4. Discussion

In this study we found 12 multi-exon conserved RPE expressed lncRNAs between the human and bovine genomes. Many have known functions, but further analysis is needed for functional characterization of RPE-specific lncRNA isoforms. All the discussed lncRNAs significantly change their expression level under culture conditions, indicating their possible role in differentiation or the maintenance of the unique function of RPE cells. The strategy we have utilized could be used in future studies to annotate functional lncRNAs in other mammalian genomes.

The low number of conserved lncRNAs we found is in good agreement with current research [44,45], however a greater level of conservation is expected between primates where there are important evolutionary advances such as the macular region of the primate retina. Only 12 out of 700 studied lncRNAs are evolutionary conserved and differentially expressed in RPE, this suggests that less than 5% of lncRNA genes are conserved in RPE in the human–bovine comparison. This estimate is close to the estimate of conserved lncRNA genes that can be traced back to the origin of tetrapods (>300 million years ago) [23]. Another study produced a large fraction of conserved lncRNAs: for 1898 human lincRNAs expressed in nine tissues across multiple individuals, orthologous lincRNA transcripts were found for 39% in the cow, 38% in the mouse, and 35% in the rat [25]. It should be noted that estimates of fractions of conserved lncRNA genes depend on different conditions of experiments and even different definitions of conserved lncRNA genes. In any case, the comparison of various estimates are suggestive of the evolution of primate-specific lncRNAs to accommodate the needs of the new eye design.

Our study has some limitations. For example, lncRNAs with a single exon were not considered for analysis as they are considered unreliable, following previous studies [2,23,27,46], and conservation was assessed based on whole genome alignment with stringent identity present. This criterion could exclude some lncRNAs that were less conserved at the nucleotide level. In this regard, it has been suggested that the secondary and tertiary structure of lncRNAs could be more important for lncRNA function rather than merely the primary sequence of nucleotides alone [47,48].

Identified conserved lncRNAs are expressed in many tissues other than the RPE, but some isoforms might have tissue-specific expression, such as the shorter NEAT isoforms in human RPE. RPE-specific isoforms indicate distinct roles in the eye. Differences in the intron–exon structure of conserved lncRNAs suggest that there might be faster evolution of these regions, when compared to protein-coding genes.

RPE cells are important in the absorption of excess light, transport of nutrients and ions, the retinoid visual cycle, and in the phagocytosis of photoreceptor outer segments. These broad functions of RPE in the eye play a major role in determining its unique gene expression. We found a small set of conserved lncRNAs that could be involved in signature RPE functions conserved across mammals. Our findings demonstrate that the vast majority of lncRNA genes expressed in RPE are not evolutionarily conserved in mammalian genomes, nevertheless there may be a few conserved lncRNAs across various mammalian taxa that could be crucially important for eye function and development. However, the fraction of conserved lncRNAs in the overall pool of lncRNA found in RPE appeared to be very small, perhaps reflecting fast and flexible adaptation of the mammalian eye to various environmental conditions.

## Figures and Tables

**Table 1:** RNA-seq datasets.

Data Set	Reads/Library	Description	Publication
Nasal, temporal, and macular regions of human retinal pigment epithelium (RPE)/choroid	2 × 100 bp Paired-end stranded	Retina and RPE/choroid from the temporal, macular, and nasal regions of four clinically normal human donor eyes	Whitmore et al. 2014 [35]
Human ARPE-19 cells	2 × 50 bp Paired end rRNA depleted	ARPE-19 cells cultured for 4 months	Samuel et al. 2017 [34]
Bovine RPE cells	2 × 50 bp Paired end rRNA depleted	Fresh native bovine RPE; bovine RPE cultured for 4 and 8 weeks	This study

**Table 2:** lncRNAs conserved between human and bovine genomes and expressed in native RPE.

	Native Retina/RPE			Bovine 4 Weeks Culture/Fresh RPE			Bovine 8 Weeks Culture/Fresh RPE			ARPE19 Cells 4D/4M			Human
Gene Name	Base Mean	Fold Change	padj	Base Mean	Fold Change	padj	Base Mean	Fold Change	padj	Base Mean	Fold Change	padj	Annotation
MSTRG.13090	6175.61	2.43	7.45 × 10^−31^	9016.25	1.87	8.01 × 10^−15^	3028.53	1.42	1.01 × 10^−3^	0.64	0.12		NEAT1
MSTRG.13096	5.99	1.64	2.18 × 10^−3^	47.69	2.79	2.44 × 10^−6^	333.50	6.99	2.04 × 10^−43^	0.64	1.31		NEAT1
MSTRG.5066	2160.63	7.28	1.38 × 10^−49^	73.54	−3.38	1.15 × 10^−11^	30.64	−3.65	5.59 × 10^−6^	166.77	−4.29	9.70 × 10^−42^	linc00982
MSTRG.5821	5222.03	−0.52	4.94 × 10^−4^	351.11	−0.21	5.20 × 10^−1^	111.81	−1.34	3.96 × 10^−4^	2399.04	−0.22	6.12 × 10^−3^	TUG1
MSTRG.2517	212.94	−2.91	5.43 × 10^−5^	555.27	−1.79	9.19 × 10^−10^	239.46	−1.70	5.02 × 10^−10^	1087.27	−0.15	2.24 × 10^−1^	LINC000094
MSTRG.1397	55.94	0.21	6.69 × 10^−1^	5.07	−4.88	1.55 × 10^−3^	18.75	−5.98	3.84 × 10^−6^	15.11	−2.33	5.31 × 10^−3^	OTX2-AS1
MSTRG.1909	130.71	0.29	2.35 × 10^−1^	17.39	−1.98	3.23 × 10^−2^	28.53	−4.03	4.62 × 10^−6^	45.83	7.83	4.05 × 10^−13^	LINC01833
MSTRG.14528	599.24	−1.30	7.77 × 10^−16^	153.68	−1.22	9.62 × 10^−5^	88.45	−0.08	8.86 × 10^−1^	0.00			MAGI2-AS
MSTRG.5719	236.41	−3.93	6.49 × 10^−20^	357.87	−5.51	5.83 × 10^−46^	151.93	−5.61	5.85 × 10^−20^	0.00			RPS27P25-202
MSTRG.19295	56.36	0.08	7.79 × 10^−1^	674.42	1.22	1.00 × 10^−3^	20.14	2.93	2.25 × 10^−5^	0.50	0.48		FTX
MSTRG.9718	54.45	−0.61	6.41 × 10^−1^				76.35	5.31	6.25 × 10^−7^	2239.60	−12.44	1.47 × 10^−42^	MEG3
MSTRG.18250	23.79	−0.25	8.50 × 10^−1^				215.43	2.81	5.71 × 10^−12^	0.00			ZFAS1

## Supplementary Materials

The following are available online at https://www.mdpi.com/2073-4425/10/3/205/s1: Figure S1: IGV–Sashimi plot; Figure S2: IGV, syntenic region; Table S1: RPE lncRNAs classification; Table S2: RPE lncRNAs Rfam; Table S3: Differentially expressed RPE lncRNAs; Table S4: qPCR confirmation of bovine lncRNA expression; Supplementary file 1.GFT bovine; Supplementary file 2.GFT human.
