# Supplementary figures and images for: Volatile Evolution of Long Non-Coding RNA Repertoire in Retinal Pigment Epithelium: Insights from Comparison of Bovine and Human RNA Expression Profiles
Source: Genes (Basel). 2019 Mar 8;10(3):205. doi: 10.3390/genes10030205 (PMC6471466; doi:10.3390/genes10030205)

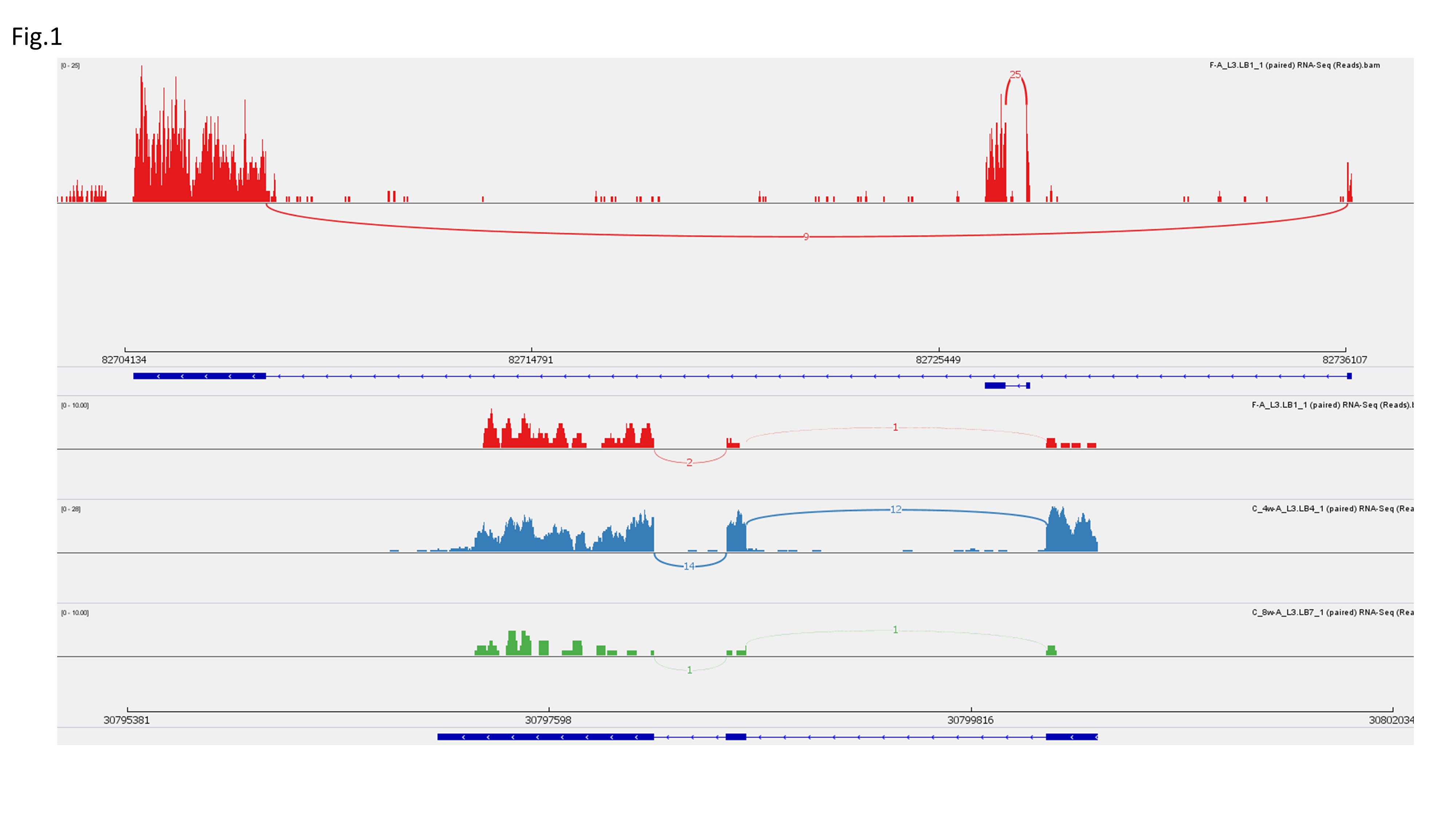

Supplement: Supplementary file 1 [file genes-10-00205-s001.zip › Slide1.PNG]

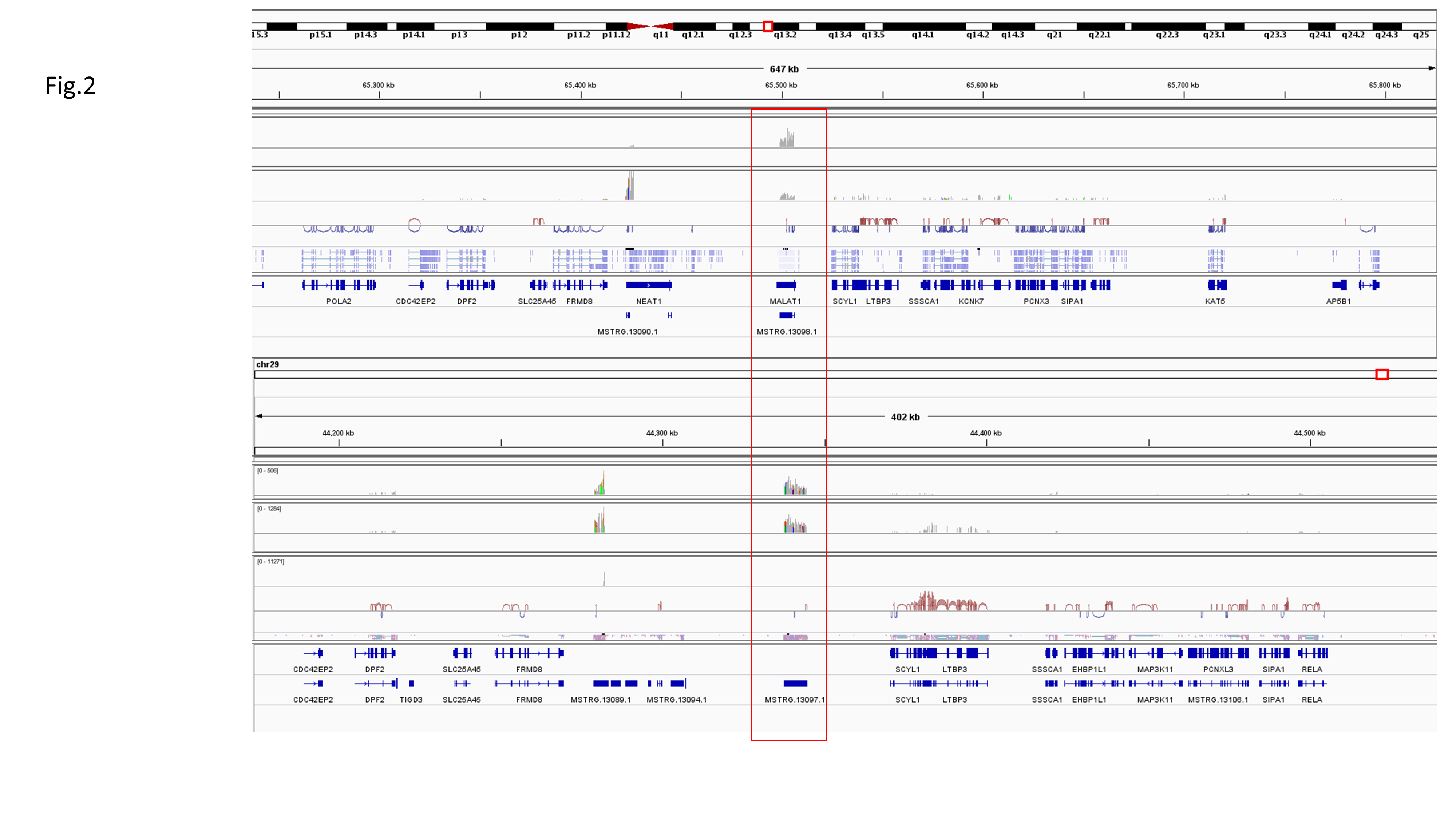

Supplement: Supplementary file 1 [file genes-10-00205-s001.zip › Slide2.PNG]
